# [Retracted] MicroRNA‑30a suppresses the proliferation, migration and invasion of human renal cell carcinoma cells by directly targeting ADAM9

**DOI:** 10.3892/ol.2024.14232

**Published:** 2024-01-15

**Authors:** Lining Jiang, Yabin Liu, Can Ma, Binghui Li

Oncol Lett 16: 3038–3044, 2018; DOI: 10.3892/ol.2018.8999

Subsequently to the publication of this paper, the authors contacted the Editorial Office to explain that, during the preparation of the data in Figs. 2C and 4D, they inadvertently included two sets of different data to represent the same experiments, and that, in fact, only the set of data panels featured on the left for each of the figure parts was intended to have been included in these figures.

The authors requested the publication of a corrigendum; however, after a subsequent investigation of the matter in the Office, as many as three pairs of overlapping data panels were identified comparing the data panels that were intended to have been included in the figures with those that were not. After careful consideration, the Editor of *Oncology Letters* has decided that the paper should be retracted from the journal on account of a lack of overall confidence in the originally presented data. Upon contacting the authors, they accepted the Editor's decision to retract the article. The Editor regrets any inconvenience caused to the readership.

